# Commentary: Plasma Metabolic Profiling of Pediatric Sepsis in a Chinese Cohort

**DOI:** 10.3389/fcell.2021.766357

**Published:** 2021-10-28

**Authors:** Tiantian Liu, Shuyun Feng, Yucai Zhang, Chunxia Wang

**Affiliations:** ^1^Department of Critical Care Medicine, Shanghai Children's Hospital, Shanghai Jiao Tong University, Shanghai, China; ^2^Institute of Pediatric Critical Care, Shanghai Jiao Tong University, Shanghai, China; ^3^Institute of Pediatric Infection, Immunity, and Critical Care Medicine, Shanghai Jiao Tong University, Shanghai, China; ^4^Clinical Research Unit, Shanghai Children's Hospital, Shanghai Jiao Tong University, Shanghai, China

**Keywords:** pediatric sepsis, metabolic profiling, metabolomics, sampling time, biomarker

## Introduction

Sepsis is a complicated and heterogeneous clinical syndrome with the overall incidence of 48 cases per 100,000 person-years (Fleischmann-Struzek et al., 2018), which is characterized by maladaptive immune and metabolic responses with ensuing organ dysfunction (Weis et al., 2017; Fitzpatrick, 2019). A hypermetabolic state occurs at the onset of sepsis, including highly amplified protein synthesis, tachypnea, tachycardia, and stress hyperglycemia (Pravda, 2014; Plummer and Deane, 2016). In 2016, Sepsis-3 in adult was defined as life-threatening organ dysfunction caused by a dysregulated host response to infection (Shankar-Hari et al., 2016). Metabolic disorders are critical for sepsis-associated organ dysfunction, which is increasingly attracting more attention (Cecconi et al., 2018). In 2020, the Surviving Sepsis Campaign guidelines for sepsis shock and sepsis-associated organ dysfunction in children refer to endocrine and metabolic therapies to maintain optimal glucose and blood calcium levels (Weiss et al., 2020). From another prospective, immunometabolism exerts its regulatory role on sepsis, and targeting the immunometabolism during sepsis offers great potential for development of novel therapeutics (Kumar, 2018, 2019; Koutroulis et al., 2019).

Serum and plasma metabolites include nucleotides, nucleosides, amino acids, glucose moieties, and more. Metabolome profiling provides a new and comprehensive perspective on a host's metabolism, which makes it a possible way to diagnose a patient or develop novel biomarkers (Wilmanski et al., 2019; Bar et al., 2020). Clinical metabolomics has been recognized as a promising tool for prediction of sepsis mortality (Wang et al., 2020). However, study on pediatric sepsis metabolic profiles is limited. Recently, Li et al. (2021) reported the serum metabolome profiling of pediatric sepsis.

## Subsections Relevant For the Subject

Li's research group reported that serum or plasma metabolite biomarkers could early predict pediatric sepsis, distinguish different pathogens, and assess severities, with a potential prospective for promoting personalized and accurate diagnosis of pediatric sepsis. Based on the comparison analysis of serum or plasma metabolome profiling in 84 cases with sepsis and 59 healthy controls from Guangzhou Women and Children's Medical Center in China, the results were predominantly carried out in the following three aspects:

First, compared with controls, the serum levels of amino acids and carbohydrates were increased, and lipids and their derivatives were decreased in patients with sepsis. Moreover, the top five decreased metabolites were lipids, while the top five increased metabolites were amino acids in non-survivors compared to survivors in septic children.

Second, Li et al. (2021) selected 9 significantly differential metabolites in 3 major bacterial species and identified that cholic acid, isovalerylglycine, and histidine were increased in patients infected with *S. aureus* vs. those with *P. aeruginosa* and *C. albicans*.

Third, correlation analysis between serum metabolic profile and blood gas analysis in sepsis cases indicated that the highly enriched oxidation of very long chain and branched-chain fatty acids pathway correlated with lactate, whereas the metabolism of methionine and linoleic acids was enriched, which correlated with bicarbonate.

## Discussion

However, we have several main issues to discuss about this study, which could be critical for drawing the conclusion.

The first issue is about the definition of pediatric sepsis. According to the inclusion criteria based on Sepsis-3 in 2016, Li et al. (2021) collected 84 sepsis cases admitted to the ICU. Sepsis-3 was developed for adult patients. Whether it is applicable to children is controversial, such as SOFA scores are changeable with age (Matics and Sanchez-Pinto, 2017). According to the International Pediatric Sepsis Consensus Conference (Goldstein et al., 2005) in 2005, pediatric sepsis is defined as the presence of the suspected or proven infection plus two or more systemic inflammatory response syndrome (SIRS), including core temperature >38.5 or <36°C; tachycardia or tachypnea, defined as mean heart or respiratory rate >2 SD above normal rate for age; abnormal high or low white blood cell counts for age or >10% immature bands, which is different from the definition of Sepsis-3 in adults. Furthermore, the management of pediatric sepsis is independent of the guideline of adult sepsis. In addition, these children included in this study were admitted to the ICU from 2015 to 2016. So, it is inappropriate from the timeline for these patients to diagnose sepsis according to Sepsis-3 published in February 2016. So, we suggest that the definition for sepsis in pediatrics is more suitable for this article.

The second issue is about the sample types and sample selection time. “Plasma” was mentioned in the title and materials, and “serum” was used in the second part of results and discussion. There is a little confusion about sample type. Though serum or plasma could be used for metabolomics analysis, authors should confirm the consistency of sample types because the levels of metabolites are closely related to sample types. Furthermore, the relationship of sample collection time with disease process was critical for the result of metabolomics analysis based on clinical samples. Though it was mentioned in the Discussion section that the collection time was the day of admittance, the interval time between sampling and sepsis diagnosis was lacking and was not discussed. In addition, underlying diseases [for example, genetic metabolic diseases (Motiani et al., 2020)] and medical prevention before ICU admission could be other confounders for the results of metabolomics analysis. So, it is necessary to describe the detailed sampling collection criteria and list the baseline characteristics of patients with sepsis.

The last is about the correlation analysis between metabolic profile and blood gas. Usually, there would be many times of blood gas analysis for patients with sepsis during PICU hospitalization. Though Li et al. (2021) reported the correlation analysis between metabolic profile and blood gas, there was no detailed information about the time point about blood gas analysis, and there was no description about whether it was the same time for blood sampling for metabolic analysis and blood gas analysis. More importantly, the results of blood gas analysis usually are different between arterial and venous blood.

In summary, data analysis based on metabolomics analysis could be simple and expected with some results, but how to understand and define these results needs more detailed information about the status of patients and blood sampling, as well as the corresponding variables enrolled into further analysis.

## Author Contributions

TL and CW wrote, reviewed, and edited the manuscript. SF and YZ reviewed the manuscript and contributed to discussion. All authors contributed to the work and approved it for publication. CW was the guarantor of this work and took responsibility for the integrity and the accuracy of this work.

## Funding

This work was supported by National Natural Science Foundation Grants (82171729), Natural Science Foundation of Shanghai (19ZR1442500), and Shanghai Municipal Education Commission-Gaofeng Clinical Medicine Grant (20171928).

## Conflict of Interest

The authors declare that the research was conducted in the absence of any commercial or financial relationships that could be construed as a potential conflict of interest.

## Publisher's Note

All claims expressed in this article are solely those of the authors and do not necessarily represent those of their affiliated organizations, or those of the publisher, the editors and the reviewers. Any product that may be evaluated in this article, or claim that may be made by its manufacturer, is not guaranteed or endorsed by the publisher.
